# Time-of-day dependence of neurological deficits induced by sodium nitroprusside in young mice

**DOI:** 10.1186/1740-3391-9-5

**Published:** 2011-06-17

**Authors:** Mamane Sani, Hichem Sebai, Naceur A Boughattas, Mossadok Ben-Attia

**Affiliations:** 1 Département de Biologie, Faculté des Sciences de Maradi, Université de Maradi, 465 Maradi, Niger; 2 Laboratoire de Biosurveillance de l'Environnement, Faculté des Sciences de Bizerte, 7021 Zarzouna, Tunisia; 3 Laboratoire de Pharmacologie, Faculté de Médecine, 5019 Monastir, Tunisia

## Abstract

Sodium nitroprusside (SNP) is widely used in pharmacological studies as a potent vasodilator or a nitric oxide donor. SNP-induced ataxic effects were assessed in mice by the Joulou-Couvoisier test. Swiss albino mice of both genders, 2-8 weeks of age, were acclimated at least for 2 weeks to 12 h light (rest span)/12 h dark (activity span). In 2 and 4 week old mice, maxima of ataxia were found following intraperitoneal administration of a dose ranging from 3 to 3.6 mg.kg^-1 ^SNP at ≈ 1 and 13 HALO (Hours After Light Onset). The sublethal toxicity was statistically dosing-time dependent (χ^2 ^test: *P *< 0.005). No rhythm was validated in neurotoxicity by cosinor analyses. At the 8^th ^week of post-natal development (PND), SNP-induced ataxia was greatest at ≈ 1 HALO (69% in males *vs*. 49% in females) and lowest at ≈ 13 HALO (21% in males *vs*. 11% in females) (χ^2 ^test: *P *< 0.00001). Cosinor analysis also revealed no statistically significant rhythm in mice injected with 3 or 3.3 mg.kg^-1^. However, a significant circadian (τ = 24 h) rhythm was detected by adjusted cosinor in 3.6 mg.kg^-1^-treated mice (*P *< 0.004). In all studied groups, SNP-induced motor impairment (expressed in %) was lower during the dark than the light phase. Furthermore, there was a non-significant gender-related difference in SNP-induced neuronal toxicity with the males more sensitive than females at every studied PND. The ataxic effects were inversely proportional to the lag time from injection and to the age of animals (with *P *< 0.05 only between 2 and 8 week old mice). These data indicate that both the administration time and age of the animal significantly affect the neurotoxic effects of SNP.

## Background

Sodium nitroprusside [Na_2_(Fe(CN)_5_NO] has been known to inorganic chemists since 1849 [1]. SNP is clinically used for lowering blood pressure in hypertension emergencies [2], for producing controlled hypotension during anaesthesia [3], and for treating acute myocardial infarction [4] and chronic heart failure [5]. However, toxic effects of this drug have been reported [6], originally ascribed to the nitroso moiety or to various decomposition products such as cyanide, thiocyanate, and nitrite. It was postulated that the iron atom of the nitroprusside complex reacts with free sulfhydryl groups (-SH) in erythrocytes and releases cyanide *in vivo *by nonenzymatic reaction [7]. Free cyanide can be converted to thiocyanate by the enzyme thiosulfate sulfurtransferase (rhodanese) that is present in various tissues [8-10] and particularly high in liver [11]. Moreover, the vasodilating effect of SNP is due to of its ability to release nitric oxide (NO) group to the vascular wall. Release of the NO is triggered by reduction of the (CN)_5_FeNO^-2 ^anion. SNP has been suggested to cause cytotoxicity through either the release of cyanide and/or nitric oxide. The major target of NO is soluble guanylate cyclase [12], but many more molecules are modified by NO or reactive oxygen species derived from NO. Other studies reveal that the *in vitro *cytotoxicity of SNP may or may not be mediated by NO [13]. Indeed, some NO donor compounds (e.g. *S*-nitroso-*N*-acetylpenicillamine, *S*-nitrosoglutathione) have been shown to protect neurons against oxidative injury and cell death caused by small molecular weight iron complexes such as ferrous citrate [14]. With this discrepancy, it is debatable whether the SNP's neurotoxicity is or is not due to the NO release.

Biological rhythms are regular and periodic phenomena existing in all living organisms [15]. In fact, most physiological functions have a rhythm with a period of approximately 24 h [15]. Desired and nondesired (toxic) effects of several chemical and physical agents also vary markedly within the 24-h period depending on the time of administration [16-19]. The rhythms in drug effects are suggested not to be due to rhythmic changes in the pharmacokinetics of drugs but rather to an endogenous rhythm in drug susceptibility resulting from a circadian rhythm controlled by an inner clock [20]. Thus, the dosing of medication at the targeted biological time with reference to circadian rhythms can result in modulation of its toxicity [21]. The presence of this phenomenon that is extremely significant for pharmacologic studies and clinical practice has been demonstrated to a considerable extent in animals [22,23]. The present study investigated dosing-time-dependent effects of SNP on motor coordination in 2-, 4-, and 8-week old mice.

## Materials and methods

### Animals and synchronization

This work was carried out at the Faculty of Sciences of Bizerte (Tunisia), Laboratory of Toxicometry and Chronobiometry. The experiments lasted from February to July, 2006. These were conducted on Swiss albino mice, 2 to 8 weeks of age that were housed 4 or 5 per cage and used in accordance with the local ethic committee of Tunis University for use and care of animals in conformity with the NIH recommendations. Animals were acclimated for at least 2 weeks prior to and during experiments [24], to controlled conditions of light cycle (12 h light-12 h dark), temperature (22 ± 2°C) and relative humidity (50-60%) with daily checks. Animals were kept in two air-conditioned rooms especially designed for chronobiologic investigations by having an inverted light regimen to explore several circadian stages during the usual diurnal work span. Luminosity was assessed using a Gossen-Panlux Electronic light meter held at the level of the cage lid and perpendicular to the incident ray from the light source. A week after birth, before the assay, young animals of each gender were distributed randomly and housed in groups of 5 (4 baby mice with 1 mother mouse) in cages. All animals were weaned at the age of 21 days. Standard food (ALMES, TN) and water were provided *ad libitum*. The desired synchronization was assessed by the quantification of prominent ultradian rhythms in 2 week old mice and prominent circadian rhythms in 4 and 8 week old mice. The study was performed on a total of 324 mice 2-8 aged weeks. Animals were randomly assigned with respect to age and gender to six groups (with 6 animals per group), each for treatment with sodium nitroprusside at one of six different circadian stages denoted as 1, 5, 9, 13, 17, and 21 HALO.

### Drug and motor toxicity

Sodium nitroprusside [Na_2_Fe(CN)_5_NO.2H_2_O] brown-red powder was purchased from E. Merck (Darmstadt, Germany). The SNP is a chemical product of synthesis that is hydrosoluble but little soluble in alcohol. Solutions were freshly prepared each experiment day by adding an adequate volume of sterile distilled water to obtain the desired concentration. Each dose was administered to mice in a fixed fluid volume (10 mL.kg^-1 ^*b.w*.).

In chronotoxicologic study the use of a high dose induces the alteration of rhythmicity referring to masking the dosing-time dependent differences. In adult mice neurotoxic effects of SNP were triggered with doses ranging from 2.5 to 5 mg.kg^-1^-a median toxic dose TD_50 _(dose inducing 50% motor inco-ordination) equal to 3.6 ± 0.5 mg.kg^-1^. Since there's a large difference of sensitivity between 2 and 8 week old mice, only neurotoxic, but nonlethal, doses were selected for this study. Then, three different doses of SNP were prepared following a geometric progression with a ratio of 1.1 between each two successive doses (3.0, 3.3, and 3.6 mg.kg^-1 ^*b.w*.). Each mouse of each age group (6 mice per time point) was administered intraperitoneally (*i.p*.) once a single dose of SNP at six pre-selected circadian stages. Behavioural neurotoxicity (ataxia) has been assessed as motor inco-ordination by traction test developed by Courvoisier et al. [25]. This simple test detects neurologic deficits in mice. For this test, each mouse is hanged from a taut-thread horizontally by its front paws. In this classical procedure, treated animals are tested for ten seconds for their ability to wind up one (or two) hind paw (s) on the thread. The test is considered positive when the treated animal is able to wind up one (or two) of its hind paws. Motor performance is measured as the percentage of animals that fall off the thread or that can not wind up their paws during the test. SNP was administrated 5 min before testing. All animals were tested simultaneously at 5, 10, 15, 20, 25, 30, 35, 40, and 45 min following injection or until complete recovery. The group end-point we used for this test was the percentage (%) of animals falling off the thread or that can not wind up their paws before 10 seconds elapsed test time. To assess the precision of the study we repeated four times each experiment with the corresponding dose.

### Statistical analysis

Data on all experimental treatment days were expressed as % of response rate in the corresponding test time. Means (m) and one standard error of the mean (SEM) or one standard deviation (SD) were calculated. Time point data were expressed as m ± SD or ± SEM, and pertinent curves were drawn. The statistical significance of differences in motor inco-ordination rate between groups was substantiated by two-way contingency tables with generalization of Chi-square test (InStat for MacIntosh GraphPad Software, San Diego, CA, USA). Time series were analyzed for circadian and other rhythms using different test periods by the Cosinor method [26]. This method approximates time series data with single cosine curves of the designated periods within the range of the dominant suspected frequencies. Thus, for each frequency, the cosinor tests and confirms, for a detected rhythm, the hypothesis of non-null amplitude with the corresponding *P *value. The best fitting (least-square method) cosine function approximating the time-series data provides parameters to characterize a rhythm, namely the mesor (M: 24 h adjusted mean), amplitude (A: half of the difference between the peak and trough of the best fitted cosine function), and acrophase (Φ: peak time location, with light onset 0 HALO used as phase reference). All rhythm characteristics were obtained with their 95% confidence limits when a rhythm was detected. This was achieved when A differed from zero (non-null amplitude *F *test) with *P *≤ 0.05. The period that corresponded to the highest amplitude (highest percentage rhythm, i.e., accounting for the greatest variance) and the lowest residual mean square (error) was considered the dominant period if *P *≤ 0.01. If the confidence limits of the acrophase for a given trial period (e.g., τ = 24 h, 12 h) are greater than ± 2 h, quantification of the acrophase and amplitude is questionable [27] because the waveform pattern of the time series is likely to deviate from a sinusoidal pattern [28]. Therefore, the significance of both conventional and chronobiologic statistics was required to validate the temporal changes as rhythms [29]. Since we conduct multiple tests (of non-null hypotheses) it is necessary to use a multiple testing correction to address the problem of the critical significance level (*P*-value cutoff). One of simplest and conservative approaches used is Bonferroni correction. This method allows adjusting of *P*-values derived from multiple statistical tests to correct for occurrence of false positives. For this method, the *P*-value of each test (provided by cosinor) is multiplied by the number of performed tests (n = 36, in this case). If the corrected *P*-value is still below the error rate, the test will be significant: Corrected *P*-value = *P*-value × 36 < 0.05.

## Results

**Figure 1:**
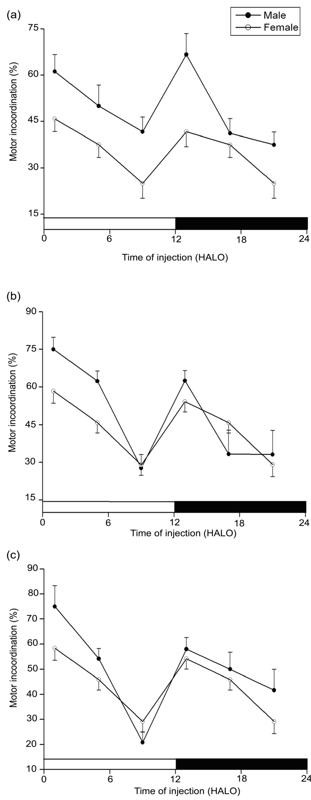
**Variations of ataxia according to the dosing time of sodium nitroprusside in two-week old male and female mice**. The three panels refer to three doses: 3 (**a**), 3.3 (**b**) and 3.6 mg.kg^-1 ^(**c**). Values are the mean ± SEM of four independent experiments. The black bar corresponds to the dark period. Maximums of motor incoordination correspond, irrespective to the gender and dose, to drug dosing at 1 and 13 HALO. χ^2 ^test validated a statistically significant difference according to dosing-time in both genders (*P *< 0.006). No statistically significant rhythm was detected by Cosinor analysis with correction for multiple testing.

**Figure 2:**
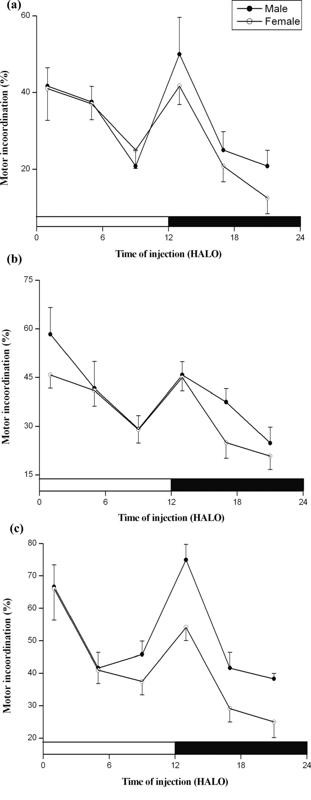
**Variations of ataxia according to the dosing time of sodium nitroprusside in four-week old male and female mice**. The three panels refer to three doses: 3 (**a**), 3.3 (**b**) and 3.6 mg.kg^-1 ^(**c**). Values are the mean ± SEM of four independent experiments. The black bar corresponds to the dark period. Maximums of motor incoordination correspond, irrespective to the gender and dose, to drug dosing at 1 and 13 HALO. χ^2 ^test validated a statistically significant difference according to dosing-time in both genders (*P *< 0.0004). No statistically significant rhythm was detected by Cosinor analysis with correction for multiple testing.

**Figure 3:**
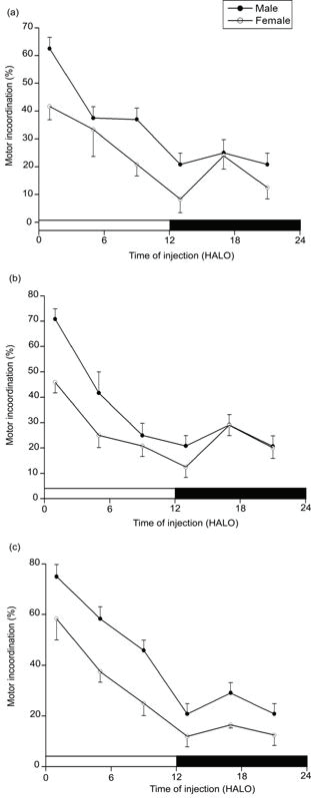
**Variations of ataxia according to the dosing time of sodium nitroprusside in eight-week old male and female mice**. The three panels refer to three doses: 3 (**a**), 3.3 (**b**) and 3.6 mg.kg^-1 ^(**c**). Values are the mean ± SEM of four independent experiments. The black bar corresponds to the dark period. Maximum and minimum of motor incoordination correspond, irrespective to the gender and dose, to drug dosing at 1 and 13 HALO, respectively. A dosing time-related effect was statistically validated by χ^2 ^test (*P *< 0.00005) in both genders. Circadian (τ = 24 h) rhythms were confirmed by Cosinor analysis with correction for multiple testing only in mice treated with 3.6 mg.kg^-1 ^(*P *< 0.05).

In 2 week old mice, sodium nitroprusside-induced locomotor impairment (ataxia) was least at 9 HALO, affecting only 30% of the animals, and was greatest at 1 and 13 HALO, affecting 62% and 56% of the animals (χ^2 ^= 16.51, *P *< 0.005), respectively (Figure 1). Similarly, in the young-weaned (4 week old) mice, drug dosing at 1 and 13 HALO resulted in highest ataxia (Figure 2). Ataxia rate differed as a function of SNP dosing-time, the difference being validated by χ^2 ^test (*P *< 0.005). In both genders, no statistically significant rhythm was detected by Cosinor analyses (Tables 1 and 2). With the three used doses (3.0, 3.3, and 3.6 mg.kg^-1^), the ataxic effects were higher in males than in females but not statistically significant. The gender-related difference in ataxia rates persisted until age 8 weeks, but their respective value was 19% and 35% lower than the rate at age 2 weeks. Moreover, with the exception of the 4 week old male mice (*P *≤ 0.01: χ^2 ^test between doses 3.0 and 3.6 mg.kg^-1^), there was no statistically significant dose-dependent ataxia at any postnatal development (PND) stage. Chronograms of ataxia at age 8 week (Figure 3) show curve patterns with the peak time located at ≈ 1 HALO (69% in males *vs*. 49% in females) and the trough time at ≈ 13 HALO (21% in males *vs*. 11% in females) (*P *< 0.00001: χ^2 ^test). No statically significant rhythm was detected in 3 and 3.3 mg.kg^-1^-treated 8 week old mice. However, a significant 24 h rhythm was detected by Cosinor analysis in mice treated with 3.6 mg.kg^-1 ^(*P *< 0.004). The behavioural neurotoxicity was inversely proportional to the age of animals; with significant (*P *< 0.05) difference only between 2 and 8 week old mice.

**Table 1:** Parameters of ultradian and circadian rhythms of sodium nitroprusside-induced motor incoordination in 2 week old mice

Dose (mg/kg)	Gender	Period τ (h)	Mesor ± SEM (% of ataxia)	Amplitude ± SD (% of ataxia)	Acrophase **±SD (min)**^*b*^	**Adjusted *P ***^*a*^
	Male	12	50 ± 4	17 ± 15	1.2 ± 120	NS
3.0		24	51 ± 4	10	9.7	NS
	Female	12	34 ± 5	19 ± 18	2.1 ± 120	NS
		24	35 ± 5	2	21.2	NS
		12	49 ± 5	26 ± 17	1.9 ± 74	NS
3.3	Male	24	50 ± 5	16	4.8	NS
		12	45 ± 4	15 ± 14	2.3 ± 120	NS
	Female	24	45 ± 4	6	1.3	NS
		12	45 ± 4	16 ± 14	2.3 ± 144	NS
3.6		24	55 ± 4	16 ± 14	2.3 ± 240	NS
	Male	12	37 ± 5	19 ± 18	1.9 ± 120	NS
	Female	24	38 ± 5	2	12.5	NS

^a ^From an _*F *_test of the rejection of the null amplitude hypothesis.

^b ^Time of maximum in fitted cosine function, in HALO.

NS: Not significant

**Table 2:** Parameters of ultradian and circadian rhythms of sodium nitroprusside-induced motor incoordination in 4 week old mice

Dose (mg/kg)	Gender	Period τ (h)	Mesor ± SEM (% of ataxia)	Amplitude ± SD (% of ataxia)	Acrophase **±SD (min)**^*b*^	**Adjusted *P ***^*a*^
	Male	12	30 ± 4	15 ± 14	1.1 ± 120	NS
3.0		24	28 ± 4	2	5.4	NS
	Female	12	33 ± 4	15 ± 14	1.2 ± 120	NS
		24	33 ± 4	12	3.2	NS
		12	49 ± 5	26 ± 17	1.9 ± 74	NS
3.3	Male	24	40 ± 4	10	8.8	NS
		12	31 ± 5	17 ± 16	1.4 ± 120	NS
	Female	24	32 ± 5	2	1.5	NS
		12	65 ± 4	18 ± 13	1.2 ± 90	NS
3.6		24	64 ± 4	16 ± 13	4.5 ± 228	NS
	Male	12	36 ± 5	16 ± 15	2.0 ± 120	NS
	Female	24	34 ± 3	4	5.0	NS

^a ^From an _*F *_test of the rejection of the null amplitude hypothesis.

^b ^Time of maximum in fitted cosine function, in HALO.

NS: Not significant

## Discussion

Although toxic effects of SNP in mice are well established, time-of-day oscillations in SNP tolerance have not been reported. Our results clearly indicate that motor activity varies as a function of the time of day after administration of SNP in mice.

Analysis of the data by means (of percent motor inco-ordination) of χ^2 ^test revealed significant (*P *< 0.005) variations of neuronal toxicity according to dosing-time of SNP in pre-weaned (2 week old) and young-weaned (4 week old) mice. Across of doses, mice treated during the second-half of daily sleep/activity phases (9, 21 HALO) showed lower motor inco-ordination, whereas mice treated during the early of sleep/activity spans (1, 13 HALO) showed much higher neurologic effects. Modulation of SNP neuronal toxicity by cosinor method revealed no significant ultradian (12 h) or circadian (24 h) rhythms, suggesting that the experimental distribution differed from a sinusoidal pattern. Raw data (Figures 1 and 2) confirm this; the curve patterns differ from regular cosine functions and it helps to explain why the cosinor provided acrophases and amplitudes with rather large confidence limits.

The two-way contingency tables revealed significant (*P *< 0.00001) time-of-day variability differences within 24-h time scale for all tested doses in young-grow-up (8 week old) animals. The most statistically significant circadian time-dependent toxicity was seen at 3.6 mg.kg^-1^-dose of SNP, where ~70% of the mice reacted negatively when treated just after light onset (1 HALO). This is contrasted with a lower neurotoxicity when mice received the same dose of SNP during the beginning of the activity span (13 HALO). Cosinor analysis revealed a significant circadian rhythm only in 3.6 mg.kg^-1^-treated mice. The maximum of SNP toxicity was at 1 HALO with regard to the raw data depicted in the chronograms and at ≈ 2.7 HALO ± 2 h with regard to cosinor analysis-determined acrophase. However, although SNP neurotoxicity exhibits a marked time-of-day dependence in 3 and 3.3 mg.kg^-1^-treated mice cosinor analysis validated no statistically significant circadian variation, indicating that the temporal patterns of drug effects are somewhat non sinusoidal. In this study, the amplitude of the 24-h SNP neurotoxicity rhythm (Table 3) was greater with the highest dose of SNP (3.6 mg.kg^-1^), implying that a greater proportion of the variability in tolerance was attributable to time-of-day dependent differences. These results suggested that the waveform of drug effects might be depending on, among other things, both dose and age of animal. The absence of rhythmicity of neurotoxic effects in pre-weaned and young-weaned mice could be explained by the immature physiological functions of some organs, as the central nervous system is still not fully developed during the first period of post-natal life [30]. Also, it has been suggested that the circadian system of foetuses and newborn pups is entrained mostly by nonphotic maternal cues during prenatal and early postnatal development [31]. Indeed, the detection of significant circadian rhythms later in 8 week old mice injected with dose 3.6 mg.kg^-1 ^would be related in part to the development of many aspects of peripheral as well as central nervous system functioning. It has been demonstrated that amplitude of the circadian component of several rhythms increases with growth and development of the mouse [32]. On the other hand, the present study showed a dose-dependent high toxicity of SNP at any PND stage in male mice comparatively to females. Indeed, it has been shown that male mice are highly sensitive to cyanide as compared to females [33].

**Table 3:** Parameters of circadian rhythms of sodium nitroprusside-induced motor incoordination in 8 week old mice

Dose (mg/kg)	Gender	Period τ (h)	Mesor ± SEM (% of ataxia)	Amplitude ± SD (% of ataxia)	Acrophase **± SD (min)**^*b*^	**Adjusted *P ***^*a*^
	Male	12	35 ± 4	8	0.42	NS
3.0		24	34 ± 4	18 ± 14	3.2 ± 108	NS
	Female	12	27 ± 4	8	2.8	NS
		24	27 ± 4	14 ± 13	2.7 ± 78	NS
		12	27 ± 4	8	2.7	NS
3.3	Male	24	26 ± 4	14 ± 12	2.0 ± 60	NS
		12	19 ± 3	3	4.5	NS
	Female	24	20 ± 3	11 ± 10	1.3 ± 90	NS
		12	41 ± 4	6	3.1	NS
3.6		24	40 ± 4	23 ± 13	3.4 ± 120	0.003
	Male	12	19 ± 3	8	1.9	NS
	Female	24	19 ± 3	20 ± 10	2.1 ± 55	0.003

^a ^From an _*F *_test of the rejection of the null amplitude hypothesis.

^b ^Time of maximum in fitted cosine function, in HALO.

NS: Not significant

Our studies therefore clearly show that mice exhibit time-of-day dependence in SNP tolerance, which is probably due to endogenous and/or exogenous periodic factors. Furthermore, temporal pattern of SNP neurotoxicity depends upon the age of animal for the same dose. Although there are many reports on the changes in the pharmacokinetic of some drugs depending on the time of administration, there is not enough information to determine whether a circadian variation is present in the pharmacokinetics. However, in animal experiments, time-dependent oscillations are observed in enzyme activities for drug metabolism in the liver [34-36]. But then, in addition, it is necessary to demonstrate a reasonable relation between the waveforms of the temporal patterns of drug levels and their effects. In our previous studies, daily variations of rhodanese, a principal enzyme of cyanide (considered as responsible of SNP toxicity) detoxification, were observed in various tissues, including the liver [9-11]. However, there was no correlation between SNP-induced neurotoxicity and the temporal variations of enzyme activities, suggesting that the toxicity of SNP is not only mediated by cyanide, and/or that the enzyme rhodanese is not the only one playing a role in cyanide detoxification. It has been reported that even the NO that is released by SNP induces neuronal toxicity [37]. That toxicity might be related to the SNP-induced oxidative effects, suggesting the involvement of reactive oxygen species (ROS) in this neurotoxicity. Indeed, Fukushina et al. [37] have demonstrated that SNP-induced neurotoxicity is attenuated by super-oxide dismutase (SOD), an enzyme well known to scavenge superoxide radicals (O_2_^●-^). However, the partial protective effect of SOD is likely due to the fact that factors other than ROS may also be involved in SNP-induced neurotoxicity. Moreover, in addition to SOD, several other anti-oxidant factors such as reduced glutathione, glutathione peroxidase, and catalase may contribute to the reduction of the increased ROS production [38,39]. The circadian peak and trough times of ataxia in 8 week old animals matched well with the observed maximum and minimum of oxidative effects after SNP (2.5 mg.kg^-1^) injection at the early light and dark spans, respectively [39]. However, the high sensitivity of male mice compared to females matched the observed high levels of rhodanese in females [9-11], indicating partially the great influence of cyanide on SNP-induced toxicity. On the other hand, these results revealed that there were opposite effects depending on the time of administration in SNP-induced neurotoxicity, illustrating that there are not only quantitative differences in drug effects.

These findings revealed that reactions to SNP were essentially similar regardless of the time-day of administration. These analyses indicate that the safest time for SNP may be the second half of the daily activity span, a time comparable to 1 pm to 7 pm for nocturnally resting human beings. However, despite the appearance of restless behaviour and ataxia at all the studied PND stages, circadian rhythms were only detected at the dose 3.6 mg.kg^-1 ^in young-grown-up mice. This phenomenon has been studied for other drugs in many animal species. These variations seem to be considered endogenous resulting from the daily variation in drug susceptibility of the target cells, which is not dependent on drug pharmacokinetics. When considering the assessed parameter in this study, the temporal variation might be probably attributed to the rhythms in the neurotransmitters, receptors, and second messengers.

In conclusion, these results indicate that there are time-dependent variations in SNP-induced neurotoxicity. The neurotoxic effect of SNP has been attributed to its production of ROS by releasing NO. However, no experimental evidence to support this interesting hypothesis is yet available. One potentially useful way of demonstrating oxidative effects (NO neurotoxicity) from SNP might be the administration of sodium thiosulfate (which may act by converting CN to thiocyante with assistance of the enzyme rhodanese) prior to the administration of SNP and the measure of ROS generation.

## Competing interests

The authors declare that they have no competing interests.

## Authors' contributions

MS performed experiments and wrote the first version of the manuscript. HS, NAB, and MBA participated in the design of the study, data analysis, and editing of the manuscript. All authors read and approved the final version of the manuscript.
